# Biomarkers and focused ultrasound: the future of liquid biopsy for brain tumor patients

**DOI:** 10.1007/s11060-021-03837-0

**Published:** 2021-10-06

**Authors:** Jordina Rincon-Torroella, Harmon Khela, Anya Bettegowda, Chetan Bettegowda

**Affiliations:** Department of Neurosurgery, Johns Hopkins University School of Medicine, 600 N Wolfe St, Phipps 118, Baltimore, MD 21128 USA

**Keywords:** Biomarkers, Focused Ultrasound, Gliomas, Liquid biopsy, Review

## Abstract

**Introduction:**

Despite advances in modern medicine, brain tumor patients are still monitored purely by clinical evaluation and imaging. Traditionally, invasive strategies such as open or stereotactic biopsies have been used to confirm the etiology of clinical and imaging changes. Liquid biopsies can enable physicians to noninvasively analyze the evolution of a tumor and a patient’s response to specific treatments. However, as a consequence of biology and the current limitations in detection methods, no blood or cerebrospinal fluid (CSF) brain tumor-derived biomarkers are used in routine clinical practice. Enhancing the presence of tumor biomarkers in blood and CSF via brain-blood barrier (BBB) disruption with MRI-guided focused ultrasound (MRgFUS) is a very compelling strategy for future management of brain tumor patients.

**Methods:**

A literature review on MRgFUS-enabled brain tumor liquid biopsy was performed using Medline/Pubmed databases and clinical trial registries.

**Results:**

The therapeutic applications of MRgFUS to target brain tumors have been under intense investigation. At high-intensity, MRgFUS can ablate brain tumors and target tissues, which needs to be balanced with the increased risk for damage to surrounding normal structures. At lower-intensity and pulsed-frequency, MRgFUS may be able to disrupt the BBB transiently. Thus, while facilitating intratumoral or parenchymal access to standard or novel therapeutics, BBB disruption with MRgFUS has opened the possibility of enhanced detection of brain tumor-derived biomarkers.

**Conclusions:**

In this review, we describe the concept of MRgFUS-enabled brain tumor liquid biopsy and present the available preclinical evidence, ongoing clinical trials, limitations, and future directions of this application.

## Introduction

From 2013 to 2017, the average annual age-adjusted incidence rate of all central nervous system (CNS) tumors in the United States was 23.79 per 100,000 people [1]. Glioblastoma (GB) represents the most common malignant primary brain tumor, with a dismal five-year survival rate of less than 7 percent [1, 2]. Therapeutic approaches for primary brain tumors are typically multimodal, with the current standard of care of GB involving maximal surgical resection followed by the Stupp protocol: chemotherapy with temozolomide (TMZ) and concomitant radiation therapy [3–6]. Yet, despite extensive research efforts, many patients still experience poor outcomes and low survival rates. Due to the diffusely infiltrative nature of brain tumors, gross total resection is often not curative but rather a measure to increase survival, alleviate neurological symptoms, and optimize the patient’s ability to tolerate subsequent post-surgical treatment [3, 4]. Profound genetic, epigenetic, and morphological heterogeneity are hallmarks of primary brain tumors. Such heterogeneity evolves over time as these tumors adapt to treatment, hindering complete tumor eradication and promoting treatment-resistant recurrences. The radiographic and clinical similarities between tumor recurrence and pseudoprogression complicate the management of brain tumor patients who too often require repeat surgeries to guide care [7]. Liquid biopsy is still constrained by the evolving understanding of tumor biomarkers and their limited representation in body fluids compared to normal tissue elements. Thus, enhancing the presence of tumor biomarkers in blood and cerebrospinal fluid (CSF) via brain-blood barrier (BBB) disruption with MRI-guided focused ultrasound (MRgFUS) is a very compelling strategy for future management of brain tumor patients. The present review describes the current research, applications, challenges, and limitations of combining MRgFUS with brain tumor biomarkers.

### The challenges of brain tumor therapy

Poor prognoses for brain tumor patients can be partially attributed to the inadequate penetration of chemotherapeutic agents into the CNS due to the selective permeability of the BBB [8].

Functionally, the BBB comprises endothelial cells, astrocyte end-feet, and pericytes, all of which are bound together via tight junctions that prevent the diffusion of most substances into the brain [9]. Transport across the BBB is typically achieved through passive diffusion of small (less than 400 daltons), non-polar lipophilic molecules or via active transport [10]. Due to this highly selective permeability, delivery and penetration of most brain tumor drugs or small-molecule therapeutics is often severely limited. Thus, nearly 98% of small-molecule drugs do not pass through the BBB [8, 11]. In primary brain malignancies, the BBB is dysfunctional, and patients have variable regions of BBB disruption, indicating abundant spatial diversity within different areas of the tumor itself [12]. The intertumor and intratumor heterogeneity plagues molecular-based brain tumor therapies and makes it difficult to ascertain the penetration and efficacy of therapeutic agents. Although most chemotherapeutic agents feature large molecular weights, TMZ is a smaller, lipophilic molecule (194 daltons). This characteristic facilitates its transport across the BBB and its ability to reach therapeutic threshold concentrations in the CSF [13]. However, due to the limited impact of TMZ on patient survival and the likelihood of TMZ-related resistance, novel delivery systems are increasingly being developed to improve brain tumor treatments [14].

### Enhancing the delivery of therapies

Potential solutions to bypassing these limitations imposed by the BBB have included transiently disrupting the BBB or improving drug delivery into brain tumors using nanoparticle/microparticle drug carriers, peptide-based drug delivery, radiotherapy, or local delivery [12]. Another approach includes using convection-enhanced delivery, a therapeutic strategy that slowly coordinates targeted delivery of drugs into the brain using surgically placed small-diameter catheters to infuse the target site and reduce distant spread [15]. Yet, the disadvantages of these various methods have precluded successful implementation in patients. Direct intracranial injection and convection-enhanced delivery are limited by the risk of surgical implantation and the difficulty in executing repeated deliveries [16]. Radiotherapy, while potentially overcoming challenges imposed by the BBB, can have variable efficacy, hamper the immune system, and damage healthy brain tissue. Chemically altering the BBB to improve disruption can lead to unforeseen off-target effects and risk healthy brain tissue [17]. Modifying therapeutics themselves can have low spatial specificity or even result in off-target effects [18]. These existing limitations, together with safety and efficacy concerns, hamper the routine use of those interventions. As a result, noninvasive strategies are becoming more appealing to implement transient and safe modifications to the BBB and improve permeability. MRgFUS has emerged as a valuable intervention for overcoming the anatomical impediments posed by the BBB given the precision afforded by concurrent MRI. To date, studies across various preclinical brain tumor models have exhibited promising results to improve access to brain tumor tissue and enhance systemic treatment efficacy [19–30].

### The use and limitations of brain tumor biomarkers

Beyond limiting therapeutic access into the brain, the low permeability of the BBB additionally hinders the release of biomarkers of brain pathology into the peripheral circulation [31]. Such molecular biomarkers can include DNA mutations, epigenetic alterations, DNA copy number alterations, protein, or microRNA expression, among others. Biomarker detection in brain tumors can be crucial in providing information on the diagnosis, prognosis, and development of individualized treatment for patients harboring targetable molecular changes, which has led to the integration of molecular marker detection in clinical practice [32]. Traditional approaches to detecting tumor-derived biomarkers include direct surgical tissue biopsy to analyze the tumor landscape. However, repeated biopsies may not be feasible due to the elevated risk of complications and morbidity for brain tumor patients. Although surgical resection or stereotactic biopsies are performed for histologic confirmation and genetic profiling, these techniques require brain surgery. Repeated tumor biopsies may be needed to track tumor development or treatment amenability and can be accompanied by severe complications such as hemorrhage or infection [33]. Stereotactic biopsy for brain tumor diagnosis carries a 5–10% risk of significant morbidity and substantial complications, as studied in meta-analyses and population-level studies [33, 34]. Therefore, repeated tumor biopsies to track treatment response, change in tumor biology, and distinguishing between recurrence and pseudoprogression are not often feasible. Noninvasive liquid biopsies are now emerging as a way to provide the same information that traditional tumor biopsies offer but in a safer modality [35, 36]. Through liquid biopsies, brain tumor biomarkers can be detected in peripheral blood or CSF (Table 1) [37–67]. This way, the evolution of a tumor and a patient’s treatment response could be tracked through a simple, minimally invasive test.

**Table 1:** Overview of studies utilizing liquid biopsy to detect circulating tumor-derived biomarkers in brain tumor patients (*n* = 31)^*^

Authors (Year)	*N*	Pathology (N)	Detection strategy	Source (control)	Biomarker	Detection rate of biomarker^**^	Relevant specific targets/mutations as biomarkers
Balaña et al. (2003) [37]	28	GB	MS-PCR	Serum (control: snap-frozen tumor, PBL)	ctDNA	34.3–53.6% (prevalence)	Methylation of MGMT, p16, DAPK, RASSF1A
Weaver et al. (2006) [38]	10	GB (6), anaplastic astrocytoma (2), anaplastic oligoastrocytoma (1), oligodendroglioma (1)	MS-PCR	Plasma (control: frozen tumor, PBL)	ctDNA	60% (concordance)	Methylation of MGMT, p16, p73, RARβ
Skog et al. (2008) [39]	25	GB	RT-PCR	Serum (control: snap-frozen tumor, exosomes from 30 normal individuals)	mRNA and miRNA in microvesicles (exosome)	28% (% detection)	EGFRvIII
Lavon et al. (2010) [40]	70	High-grade astrocytomas (41), oligodendroglial tumors (29)	PCR-based LOH, MS-PCR	Serum (control: FFPE tumor, PBL, genomic DNA from healthy donors serum)	cfDNA and ctDNA	30–83% (concordance)	LOH Chromosome 1p LOH Chromosome 19q LOH Chromosome 10q Methylation status of MGMT and PTEN
Saratsis et al. (2012) [41]	10	DIPG	Protein profiling by mass spectrometry	CSF (control: CSF from healthy and non-DIPG tumor age-matched individuals). Tumor, brain tissue, urine, and blood analyzed in smaller cohort	Tumor-secreted proteins	60–90% (% detection in CSF)	DDAH1, CypA
Boisselier et al. (2012) [42]	80	Glioma	Digital PCR	Plasma (control: plasma from healthy individuals)	ctDNA	60% (% detection)	IDH1^R132^^H^
Majchrzak-Celińska et al. (2013) [43]	33	Metastatic CNS cancer (10), astrocytoma (8), GB (7), meningioma (6), gliosarcoma (2)	MS-PCR	Serum (control: tumor, leukocytes from healthy individuals, brain fragment from a hematoma case)	ctDNA	68.75–93.75% (concordance)	Methylation of MGMT, RASSF1A, p15INK4B, p14ARF
Bettegowda et al. (2014) [44]	41	Glioma (27), medulloblastoma (14)	Digital PCR-based technologies: SafeSeqS, PCR-Ligation	Plasma (control: frozen or FFPE tumor and DNA from non-neoplastic cells of the same patients)	ctDNA	<50% for medulloblastoma; <10% for glioma (% detection)	Glioma mutations: IDH1, TP53, EGFR, PIK3CA, PTEN Medulloblastoma mutations: CTNNB1, PTCH1, KDM6A, MLL2, PTEN, TP53
Macarthur et al. (2014) [45]	11	HGG preradiotherapy (11), HGG postradiotherapy (8)	Telomere activity-based assays and immunofluorescence	Peripheral blood (control: peripheral blood from healthy volunteers)	CTC	8–72% (% detection postradiotherapy - preradiotherapy)	EGFR (for selected samples)
Müller et al. (2014) [46]	141	GB	IHC, FISH, qPCR	Peripheral blood (control: FFPE tumor, peripheral blood from healthy volunteers and carcinoma-derived brain metastases)	CTC	20.6% (GFAP % detection) 73.5% (concordant presence or absence EGFR amplification)	GFAP, EGFR gene amplification (for selected samples)
Sullivan et al. (2014) [47]	33	GB	CTC-iChip, STEAM-antibody cocktail, single-cell analysis	Peripheral blood (control: peripheral blood from healthy volunteers, FFPE tumor)	CTC	39% (% STEAM-positive cells detection) Approx. 100% (concordance of SERPINE1, TGFB1, TGFBR2, and VIM)	STEAM (Sox2, Tubulin beta-3, EGFR, A2B5, c-MET) Elevated expression SERPINE1, TGFB1, TGFBR2, and VIM. EGFR amplification in one patient with metastases
Pan et al. (2015) [48]	10	Solid brain tumors (7), leptomeningeal disease (3)	Targeted amplicon sequencing, ddPCR	CSF and plasma (control: tumor)	ctDNA	85.7% (% of detection in solid brain tumors)	EGFR, KRAS, NRAS, BRAF, AKT1, NF2
Wang et al. (2015) [49]	35	Primary CNS malignancy	Targeted sequencing followed by WES	CSF (control: frozen or FFPE tumor, matched normal tissues)	ctDNA	74% (% detection)	TP53, IDH1, TERT promoter, NF2, PIK3R1, PTCH1, PTEN
De Mattos-Arruda et al. (2015) [50]	12	BMBC (6), GB (4), BMLC (2)	Targeted capture massively parallel sequencing, ddPCR	CSF (control: fresh frozen tumor, PBL)	ctDNA	58% (sensitivity)	EGFR, PTEN, ESR1, IDH1, ERBB2, FGFR2
Gao et al. (2016) [51]	31	Primary glioma	SE-iFISH	Peripheral blood (control: FFPE tumor, surrounding tumor-free tissues, peripheral blood of healthy individuals)	CTC	77% (% detection)	Chromosome 8 polyploidy
Pentsova et al. (2016) [52]	53	Brain metastasis (41), primary brain tumor (12)	MSK-IMPACT (NGS-based tumor sequencing assay)	CSF (control: matched and unmatched normal samples and CSF of patients with cancer without CNS involvement)	ctDNA	63% (brain metastasis), 50% (primary brain tumors), 0% (controls)(% detection)	MSK-IMPACT captures protein-coding exons of 341 cancer-associated genes as well as 33 introns in 14 recurrently rearranged genes
Huang et al. (2017) [53]	12	Diffuse midline glioma	Sanger sequencing, mutation-specific PCR	CSF (control: fresh frozen or FFPE tumor, CSF from a child with hydrocephalus)	ctDNA	83% (% detection ctDNA), 66.7% (% detection of H3.3K27M)	H3K27M
Akers et al. (2017) [54]	111	GB	RT-PCR	CSF (control: tumor, non-oncologic CSF samples)	miRNA	67% (sensitivity cisternal CSF), 28% (sensitivity lumbar CSF)	miRNA signature: hsa-miR-21-5p, hsa-miR-218-5p, hsa-miR-193b-3p, hsa-miR-331-3p, hsa-miR-374a-5p, hsa-miR-548c-3p, hsa-miR-520f-3p, hsa-miR-27b-3p, hsa-miR-30b-3p
Schwaederle et al. (2017) [55]	152	Primary brain tumor	NGS	Plasma (control: tumor from selected samples)	ctDNA	32% (% detection)	54/68/70 genes panels
Figueroa et al. (2017) [56]	71	GB	RT-PCR	CSF (control: snap-frozen tumor tissue)	Extracellular vesicles RNA	61% (sensitivity)	Wild-type EGFR and EGFRvIII
Manda et al. (2018) [57]	96	HGG	RT-PCR	Serum (control: tumor in RNA*later*, serum from other neurological diseases and matched healthy volunteers)	Exosome RNA fraction	81.58% (sensitivity)	EGFRvIII
Martínez-Ricarte et al. (2018) [58]	20	Diffuse glioma	Target exome sequencing, ddPCR	CSF (control: fresh or FFPE tumor, PBL)	ctDNA	85% (concordance of gene mutations in CSF with tumor mutations)	IDH1, IDH2, TP53, ATRX, TERT, H3F3A, HISTIH3B
Santangelo et al. (2018) [59]	111	Glioma (100), brain non-glial metastases (11)	RT-PCR	Serum (control: serum of healthy controls)	miRNA	57–89% (sensitivity in GB versus healthy controls) 60–84% (sensitivity in HGG versus healthy controls)	miR-21, miR-222, miR-124-3p
Juratli et al. (2018) [60]	38	TERTp-mutant/IDH wild-type GB	Ion Torrent NGS system, ddPCR	CSF, plasma (control: snap-frozen tumor, CSF of patients with TERTp wildtype, brain metastases or hydrocephalus)	ctDNA	92.1% (sensitivity in CSF), 7.9% (sensitivity in plasma)	TERTp
Panditharatna et al. (2018) [61]	48	Diffuse midline glioma	ddPCR	CSF, plasma (control: frozen tumor, CSF and plasma from healthy pediatric subjects)	ctDNA	88% (% detection in CSF and plasma)	H3K27M
Miller et al. (2019) [62]	85	WHO grade II (13), WHO grade III (26), WHO grade IV (46)	MSK-IMPACT	CSF (control: FFPE tumor, unmatched normal samples, CSF from patients with non-malignant neurological conditions)	ctDNA	49.4% (% detection)	TERT, TP53, IDH1, CDKN2A, CDKN2B, EGFR, ATRX, PIK3CA, MET, PDGFRA
Pan et al. (2019) [63]	57	Brainstem glioma	Deep sequencing using a 68 NGS gene panel	CSF (control: fresh tumor, matched blood)	ctDNA	100% (sensitivity of mutation detection in CSF), 38% (sensitivity of mutation detection in plasma)	H3F3A, TP53, ATRX, PDGFRA, FAT1, HIST1H3B, PPM1D, IDH1, NF1, PIK3CA, ACVR1
Zhang et al. (2019) [64]	95	GB	qPCR	Serum (control: serum from healthy controls)	miRNA	77.89% (sensitivity)	miR-100
Estival et al. (2019) [65]	93	GB (not all 93 samples available for the analysis)	MS-PCR, PYR	Blood and plasma (control: FFPE tumor, non-tumor brain tissue, colon tissue, PBL)	ctDNA	31% (MS-PCR blood sensitivity), 38% (PYR plasma sensitivity)	MGMT methylation
Mueller et al. (2019) [66]	15	DIPG	WES, WGS, RNAseq, ddPCR	Plasma (control: paired tumor, commercially purchased RNA brain controls)	ctDNA	92% (% detection of H3K27M in mutant cases)	H3F3A, HISTIH3B, H3K27M, ACVR1, PIK3R1, PPM1D, TP53, ATRX
Ma et al. (2020) [67]	21	Non-small cell lung cancer brain metastasis	NGS using the Ion system	CSF, peripheral blood (control: 5 brain tumors)	ctDNA	95.2% (ctDNA detection in CSF), 23.8% (ctDNA detection in peripheral blood)	EGFR, KIT, PIK3CA, TP53, SMAD4, ATM, SMARCB1, PTEN, FLT3, GNAS, STK11, MET, CTNNB1, APC, FBXW7, ERBB4, KDR

*BMBC* Brain metastases from breast cancer, *BMLC* Brain metastases from lung cancer, *cfDNA* Cell-free DNA, *CNS* Central nervous system, *CSF* Cerebrospinal fluid, *CTC* Circulating tumor cells, *ctDNA* Circulating tumor DNA, *ddPCR* Droplet digital PCR, *DIPG* Diffuse intrinsic pontine glioma, *ELISA* Enzyme linked immunosorbent assay, *FFPE* Formalin-fixed paraffin-embedded, *FISH* Fluorescence in situ hybridization, *FLIM* Fluorescence lifetime imaging microscopy, *GB* Glioblastoma, *GFAP* Glial fibrillary acidic protein, *HGG* High-grade glioma, *IHC* Immunohistochemistry, *LC–MS* Liquid chromatography-mass spectrometry, *LOH* Loss of heterozygosity, *MALDI-TOF MS* Matrix-assisted laser desorption/ionization-time of flight mass spectrometry, *miRNA* Micro RNA, *mRNA* Messenger RNA, *MSK-IMPACT* Memorial Sloan Kettering-integrated mutation profiling of actionable cancer targets, *MS-PCR* Methylation-specific polymerase chain reaction, *NGS* Next-generation sequencing, *PBL* Peripheral blood lymphocytes, *PYR* Pyrosequencing, *qPCR* Quantitative real-time polymerase chain reaction, *RNAseq* RNA sequencing, *RT-PCR* Reverse transcription polymerase chain reaction, *SE-iFISH* Subtraction enrichment and immunostaining-fluorescence in situ hybridization, *WBC* White blood cells, *WES* Whole exome sequencing, *WGS* Whole genome sequencing, *WHO* World Health Organization. ^*^This is a representative selection of studies in the brain tumor biomarkers field that is focused on their clinical results, and does not intend to cover all the body of literature available on this topic. Especially, studies with incomplete controls were excluded. ^**^Detection rate of biomarker includes sensitivity, concordance, or % detection of specific biomarker depending on the information provided by the original manuscript. When several biomarkers were studied, the lowest and highest values are represented as a range

### The evolution of liquid biopsy and biomarker detection in cancer

**Fig. 1:**
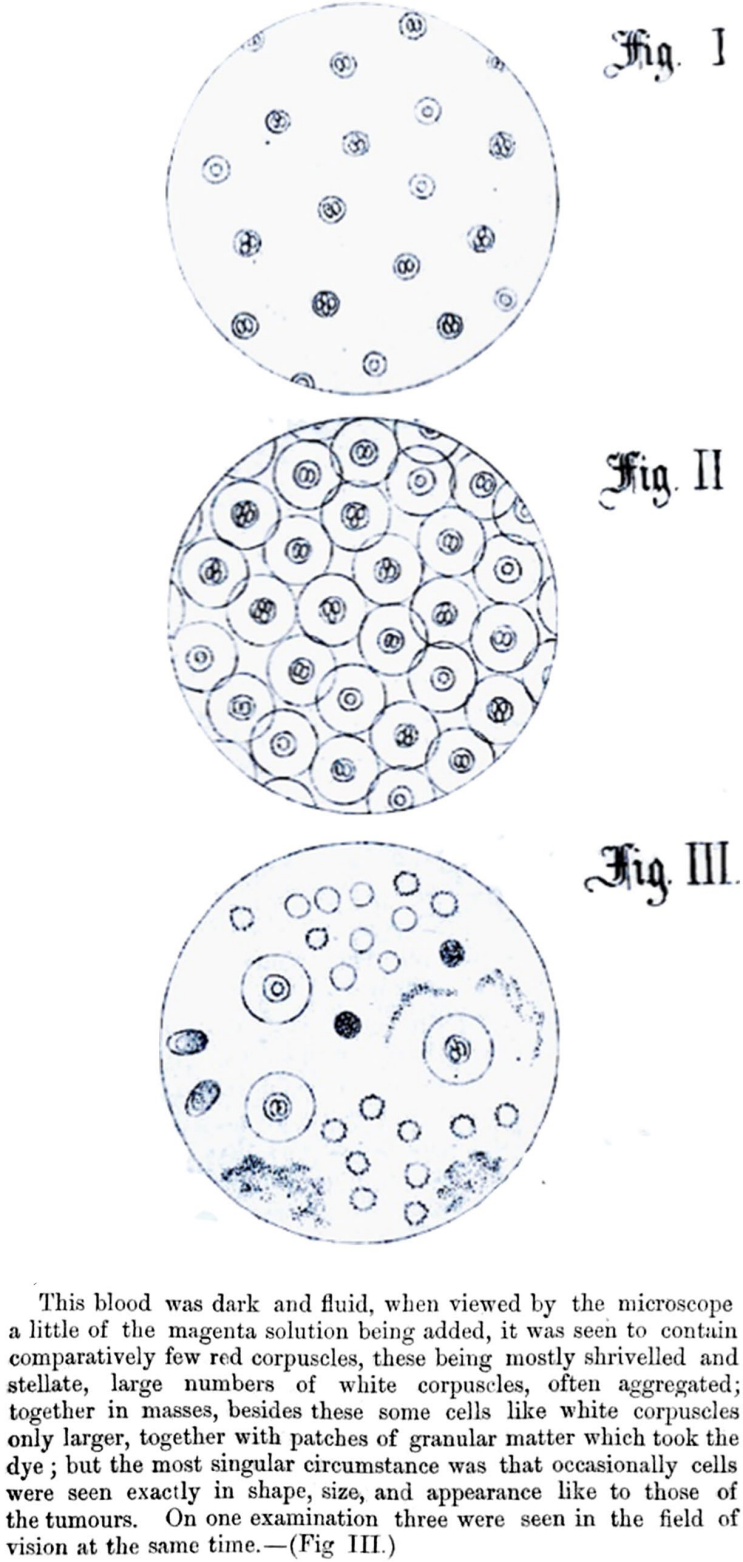
Representation of Figures I, II and III and extract from the original manuscript from *Ashworth TR. A Case of Cancer in Which Cells Similar to Those in the Tumours Were Seen in the Blood after Death. Med J Aust. 1869;14:146–147.* A 38-year old man who died of “marasmus” was found to have many subcutaneous nodules. The nodules were made of a jelly-like substance. Fig I and Fig II represent the cells that Ashworth observed in the subcutaneous tumors. Fig III represents Ashworth’s observation of cells in the blood that had an appearance similar to those of the subcutaneous tumors, together with other blood “corpuscles” as he describes in the text. This is considered the first description of circulating tumor cells in the English literature. These images are courtesy of the University of Melbourne Archives and are reproduced with their permission and permission from the Medical Journal of Australia

Liquid biopsy refers to detecting components in human fluids that are pathologically derived (e.g., tumor-derived). Common analytes of liquid biopsies for cancer include circulating tumor cells (CTC), cell-free DNA (cfDNA), and circulating tumor DNA (ctDNA) [68]. ctDNA is DNA detectable in circulation thought to be released during tumor cell apoptosis or necrosis [44]. Contrarily, cfDNA may include both germline and ctDNA. The first report of CTC is attributed to Thomas Ashworth in 1869, who described tumoral cells in the blood of a patient with metastases (Fig. 1) [69]. It was not until 1948 that Mandel et al. reported the presence of cfDNA as non-cell-bound nucleic acids in the blood of cancer patients [70]. But, it would not be until 1994 that ctDNA was distinguished from cfDNA by proving the presence of cancer-specific mutations [71, 72]. Likewise, reliable ctDNA measurements still had to wait until the late 2000s [73]. And detection is still a significant challenge: one mL blood sample contains a diminutive amount of cfDNA, of which sometimes less than 0.01% is ctDNA, especially in early cancer stages [74].

Although ctDNA and cfDNA are the most common targets of liquid biopsies, other studied analytes include proteins, mRNA, miRNA, or extracellular microvesicles [68]. Each of those analytes is present in a minimal quantity in human blood or CSF. Consequently, highly sensitive and specific extraction and amplification methods are necessary to make liquid biopsy a reality. Over the past decade, the incredible technical evolution has led to several clinical trials where ctDNA detection showed a correlation with response to treatment or recurrence in non-CNS cancers. Thus, it is already becoming an effective surveillance and follow-up technique in tumors such as breast and lung cancer [75, 76]. The same application in brain malignancies is constrained by specific limitations related to the nature and localization of CNS tumors.

Several publications have shown the possibility of detecting circulating brain tumor biomarkers [37–67]. A summary can be found in Table 1. Even though the BBB is somewhat disrupted in higher grade gliomas, it remains a prominent obstacle to tumor biomarker release into the bloodstream [77]. In fact, ctDNA is detectable in less than 10% of patients with gliomas compared to >75% of patients with other solid tumors, including advanced pancreatic, ovarian, colorectal, bladder, gastroesophageal, breast, melanoma, hepatocellular, and head and neck cancers [44]. These limitations point to an urgent clinical need to explore alternative approaches that can noninvasively enhance the release of brain tumor-derived biomarkers into the peripheral circulation. The intersection of low-intensity pulsed MRgFUS and liquid biopsies has emerged as a means to improve access to brain tumor biomarkers by inducing transient BBB opening [78–81].

### The role of focused ultrasound in brain tumors

Focused ultrasound has been established as a BBB disruption technique to ablate tissue or enhance drug delivery. High-intensity focused ultrasound (HIFU) is used for brain tumor ablation by significantly increasing tissue temperatures using ultrasound energy [82, 83]. This energy is passed through and distributed over the skull. The precise targeting of this energy to the tumor tissue is accomplished via stereotactic, steerable systems like MRgFUS [84, 85]. On the other hand, low-intensity pulsed ultrasounds are a potentially safer and quicker method in which a lower intensity of ultrasound energy is used to prevent tissue damage. Biologically, low-intensity FUS increases the permeability of the BBB through the strategic use of intravenous microbubbles [82]. The ultrasound waves cause a physical cavitation effect in the microbubbles when those pass through the FUS target region [82]. The microbubbles expand until inertial cavitation and eventually collapse, thereby mechanically disrupting the BBB via cellular membrane perforation or blood-tissue permeation [86, 87]. Nevertheless, despite such promising effects, FUS must be precisely controlled. Parameters such as the exposure frequency, microbubble diameter, pulse-repetition frequency, and burst duration can impact the extent of BBB disruption and the penetration of therapeutic agents into the brain [87]. Therefore, low-intensity FUS combined with microbubbles has emerged as an attractive approach to safely and focally induce mechanical disruptions in the BBB to improve therapeutic molecule delivery to the brain. Ongoing preclinical trials for various neurological impairments, including brain cancers [88], Alzheimer’s disease [89], and Parkinson’s disease [90], have set the foundation for the clinical translation of FUS. Human clinical trials are also determining the feasibility of this technique in various patient groups [91–94]. For example, Lipsman and colleagues demonstrated BBB opening without adverse effects after microbubble treatment with MRgFUS in 5 patients with Alzheimer's [91]. Mainprize and colleagues tested the use of MRgFUS with concomitant TMZ or liposomal doxorubicin in 5 high-grade glioma patients [92]. Both groups considered gadolinium enhancement at the sonication site as a confirmation of BBB opening. The new area of enhancement in MRI resolved overtime (24 h), indicating the reversibility of the BBB disruption [91, 92]. Employing MRgFUS to disrupt the BBB and thereby facilitate the release of brain tumor biomarkers may be an effective way to amplify the detection of these biomarkers in liquid biopsies in a noninvasive, spatially, and temporally controlled manner.

## Focused ultrasound and liquid biopsy

The combination of FUS and biomarkers was initially proposed by D’Souza et al. for human colon cancer lines in 2009 [95]. Many subsequent studies were based on the in vitro and in vivo application of HIFU in non-brain cancer tumors. For example, Chevillet et al. enhanced the release of tumor microRNA in a rat prostate cancer model [96]. D’Souza et al. increased the protein biomarkers in patients with uterine fibroids that underwent ablation by MRgFUS [97]. However, the high intensity of HIFU prevented the early application of this technique to the delicate brain tissue. Lower-intensity pulsed FUS with microbubbles have been used to study FUS and brain tumor biomarkers [78–81]. Table 2 summarizes several relevant manuscripts on FUS-enhanced liquid biopsy for brain tumors. This application has been partially explored in murine models. In 2018, Zhu et al. proposed that FUS combined with microbubbles induced disruption of the BBB that allowed both increased import and export of analytes between the brain tissue and the circulation in two orthotopic GB murine models [78]. The investigators implanted orthotopic enhanced green fluorescent protein (eGFP)-transfected human or murine glioma cells (U87 and GL261) and treated them with US-guided FUS or MRgFUS. Quantitative polymerase chain reaction (qPCR) was used to evaluate eGFP mRNA in mice blood four minutes after sonication. Circulating eGFP mRNA was significantly higher in the treated groups compared to the controls. In their proof of concept, the investigators used relatively high acoustic pressures (1.52–3.82 MPa), which consequently caused frequent FUS-induced hemorrhage at the tumor sites. Interestingly, there was no difference in biomarker release between different pressures at this higher range. The same team presented a follow-up study in 2020, now evaluating the optimal sonication pressure at which increased biomarker detection was achieved without causing hemorrhage in a murine GB model [79]. Similarly, they implanted orthotopic eGFP-transduced GL261 murine GB cells and treated them with MRgFUS and microbubbles when the tumor reached 2 mm. The researchers compared different peak negative pressure levels, used eGFP mRNA plasma levels as their biomarker of choice, and followed complications with MRI before and after sonication. A 2000- and 8000- fold increase in plasma eGFP mRNA was achieved at the higher pressures (1.29 MPa and 1.58 MPa). The sonication pressure for optimal biomarker release and low risk of hemorrhage was 0.59 MPa, reaching a 55 to 221-fold increase in eGFP mRNA compared to the controls. In their study, increased tumoral and peritumoral enhancement was associated with increased biomarker release but also post-sonication hemorrhage. To further evaluate their findings in larger animal models, the same group presented a porcine study by Pacia et al. in 2020 [80]. The researchers assessed the BBB opening in healthy pigs with MRgFUS. Increased contrast enhancement in post-sonication MRI and increased K_trans_ of gadolinium confirmed the BBB opening. Given the lack of pathology in their porcine model, their selected biomarkers were glial fibrillary acidic protein and myelin basic protein. After sonication, the researchers showed an increased concentration of those brain-specific biomarkers without major clinical or histological brain tissue damage. These summarized preclinical studies in animal models set the foundation for human clinical trials. There may be an optimal sonication pressure in patients that allows biomarker release with a low risk of hemorrhage like in the animal models. Likewise, higher sonication pressures may increase the risk of hemorrhage while the biomarker release plateaus.

**Table 2:** Overview of studies implementing focused ultrasound-enhanced liquid biopsy for brain tumors (*n* = 4)

Authors (Year)	Model (*N*)	Detection strategy	Source	Biomarker	Specific Targets	Outcome	Complications	Change in BBB permeability	FUS type	Specifications
Zhu et al. (2018) [78]	Mouse U87 and GL261 - 21 (6 control, 15 treated)	qPCR	Blood samples—terminal cardiac puncture	Tumor-specific eGFP mRNA	N/A	Levels of circulating eGFP mRNA were significantly higher in the FUS-treated mice	Brain hemorrhage was observed in the majority of cases	Assessed by contrast-enhanced MRI	Ultrasound imaging-guided FUS (VIFU 2000; Alpinion US Inc) and MRgFUS system (Sonalleve V2, Profound Medical Inc) with a 256-element phased array transducer mounted to a five-axis robot positioner	US-guided FUS parameters: frequency = 1.5 MHz, peak negative pressure = 3.82 MPa, pulse length = 10 ms, pulse repetition frequency = 1 Hz, duration = 30 s at each location, 4 locations for each tumor MRgFUS parameters: frequency = 1.44 MHz, peak negative pressure = 1.52, 2.74, and 3.53 MPa, pulse length = 10 ms, pulse repetition frequency = 1 Hz, duration = 2 min
Zhu et al. (2020) [79]	Mouse GL261 - 20 (5 control, 15 treated)	qPCR	Blood samples—terminal cardiac puncture	Tumor-specific eGFP mRNA	N/A	FUS resulted in greater plasma eGFP mRNA level compared to controls	Microhemorrhage density at 0.59 MPa was lower than at higher acoustic pressures	Increased contrast-enhancement T1-weighted MRI	MRgFUS system (Sonalleve V2, Profound Medical Inc) with a 256-element phased array transducer mounted to a five-axis robot positioner	MRgFUS parameters: frequency = 1.44 MHz, peak negative pressure = 0.59, 1.29, and 1.58 MPa, pulse length = 10 ms, pulse repetition frequency = 1 Hz, duration = 240 s
Pacia et al. (2020) [80]	Porcine normal Brain - 16 (two cohorts of 8)	ELISA	Blood samples	Brain-specific biomarkers (GFAP, MBP)	GFAP, MBP	FUS induced successful BBB opening in 7/8 pigs; FUS significantly increased plasma concentration of brain-specific biomarkers	No brain tissue damage or hemorrhage was detected	Increase in K_trans_ of the targeted brain site compared to the contralateral side (*p* = 0.0053). Increased contrast-enhancement T1-weighted MRI	MRgFUS (Image Guided Therapy, Pessac and Imasonics)	MRgFUS parameters: frequency = 0.65 MHz, peak negative pressure = 1.5 MPa, pulse length = 10 ms, pulse repetition frequency = 1 Hz, duration = 3 min
Meng et al. (2021) [81]	Human GB (9)	Qubit dsDNAHS Assay (Invitrogen) and ddPCR Nanoscale flow cytometry ELISA	Blood samples	cfDNA Extracellular vesicles (NCAM and L1CAM) s100b	IDH1 R132H mutation in one patient	2.6, 3.2, and 1.4-fold increase in cfDNA, NDEV, and S100b, respectively; two-fold to three-fold increase in IDH1 R132H mutant copies after MRgFUS	No serious adverse events	Increased contrast-enhancement T1-weighted MRI	ExAblate Neuro hemispheric device (InSightec, Israel) coupled with GE 3-Tesla MRI	Less than 20 sonication points. Other characteristics not specified

*BBB* Blood–brain barrier, *cfDNA* Circulating free DNA, *ddPCA* Droplet digital PCR, *ELISA* Enzyme linked immunosorbent assay, *FUS* Focused ultrasound, *GB* Glioblastoma, *GFAP* Glial fibrillary acidic protein, *IDH* Isocitrate dehydrogenase, *MBP* Myelin basic protein, *MRgFUS* Magnetic resonance-guided focused ultrasound, *mRNA* Messenger RNA, *NDEV* Neuron-derived extracellular vesicles, *qPCR* Quantitative polymerase chain reaction

## Clinical translation for brain tumors

In 2021, Meng and colleagues presented one of the first published evaluations of cfDNA detection enriched by MRgFUS in patients with pathologically confirmed GB [81]. In their prospective single-arm trial of GB patients, the authors treated nine patients with serial transcranial low-frequency MRgFUS and adjuvant TMZ combination. Their selected biomarkers were plasma cfDNA, neuron-derived extracellular vesicles (NDEV), and brain-specific protein S100b. NCAM and L1CAM surface proteins were used as surrogates for NDEV. Blood samples were collected on average 34 min after the last sonication. After sonication, the authors showed a 2.6, 3.2, and 1.4-fold increase in cfDNA, NDEV, and S100b, respectively. As anecdotal evidence, a two-fold to three-fold increase of IDH1-R123H mutant copies was detected by droplet digital PCR in plasma after sonication of the only patient with an IDH mutation. No major complications were reported. Despite the many limitations noted in their manuscript, Meng et al. set the basis for applying MRgFUS-based liquid biopsy in patients with GB.

There are several completed and active clinical trials for the independent study of biomarkers in brain tumors or the safety of MRgFUS. For example, The Mayo Clinic is building a biorepository of CSF samples to analyze biomarkers (NCT04692324). Comparatively, the University of Maryland is assessing the safety and feasibility of ExAblate BBB disruption for treating high-grade gliomas in the context of standard chemotherapy (NCT03322813).

Several other groups are following, and we are awaiting the results of vital clinical trials at the forefront of combining both liquid biopsy and FUS. For example, the BRAINFUL (BRAIN Tumor Focused Ultrasound-enabled Liquid Biopsy) Trial (NCT04940507) is a prospective, single-center, single-arm trial that hopes to enhance the detection of ctDNA by MRgFUS in glioma patients while understanding the changes in ctDNA over time. The BRAINFUL Trial, conducted by Dr. Lozano in Toronto, will analyze cfDNA in CSF and blood of patients that undergo tumor ablation with MRgFUS. Additionally, several grants have been recently awarded by the NIH to promote solid advances in this field. Amongst others, research teams at Washington University (1R01EB030102-01), Massachusetts General Hospital (5R01CA239078-02), and our team at Johns Hopkins, in collaboration with the University of Maryland (1R21NS113016-01), are working to revolutionize the detection and monitoring of brain tumors with the development of FUS-enabled liquid biopsies.

## Current limitations

The initiatives mentioned above will establish the foundation to translate FUS-enhanced liquid biopsy to the management of brain tumor patients. Those initiatives aim to overcome several current limitations. As described, available preclinical studies have used artificial biomarkers such as eGFP, which may not fully recapitulate the release of tumor associated biomarkers in the clinical setting. Additionally, terminal blood extractions shortly after sonication in animal experiments prevent the evaluation of FUS-induced biomarker release over time. Further studies are needed to assess the optimal sonication pressures and microbubble parameters for human brain tumor biomarker release while preserving safety. Evaluating biomarker release over time and with the influence of treatments such as radiation and chemotherapy is also paramount to understand the feasibility of FUS-enabled liquid biopsies. Tumor type, density, anatomical location, depth, skull physiology, surrounding vascular structures, and tumor-specific biomarkers may all require different parameters for optimal application of this evolving technique.

The clinical application of liquid biopsies for cancer requires rigorous assay standardization, automatization, and technical and clinical validation to analyze a large volume of samples. At the same time, one of the main limitations for the brain tumor application of MRgFUS is the frailty of the brain parenchyma. Another consideration is that current MRgFUS systems are slow, expensive, and require customization. Given the need for such specialized technology, the economic burden of current detection strategies limits its uptake. With eagerness, the scientific community works to provide a faster, more reliable, and more affordable technology. Several academic and private institutions, such as the European Liquid Biopsy Society or the International Liquid Biopsy Standardization Alliance, coordinated by the Foundation for the NIH, are working to accelerate those much needed technological advances.

Finally, ongoing clinical trials are often centered on advanced or recurrent brain tumors. Still, as knowledge and techniques progress, the goal is to detect early-stage cancers and improve the sensitivity to a point where early detection of recurrences precedes radiographic recurrence and promotes early intervention for improved survival in brain tumor patients.

## Conclusion

Many advances on liquid biopsies and MRgFUS for brain tumors have emerged over the past years. Discovering the possibilities of each independent method allowed us to explore the concept of FUS-enabled liquid biopsy. Understanding both techniques' biological and technological limitations gives us clear goals to overcome over the next decade. Some crucial aims are improving ctDNA detection methods and optimizing FUS settings to maximize biomarker release depending on the pathology and tumor location. Active research endeavors and clinical trials are underway. MRgFUS-enabled liquid biopsy is a revolutionary research field that promises a radical advance in diagnosing and monitoring brain tumors.

## Data Availability

The authors confirm that the data supporting the findings of this study are available within the article.
